# Innovating military suicide prevention: learnings from the Australian Defence SafeSide Project

**DOI:** 10.3389/fpsyt.2026.1757232

**Published:** 2026-03-13

**Authors:** Kylie Druett, Sarah Donovan, Jennifer Harvey, Dan Mobbs, Annamarie Bailey, Melanie Clark, Anthony R. Pisani

**Affiliations:** 1 Mental Health and Wellbeing Branch, Australian Department of Defence, Majura, ACT, Australia; 2 SafeSide Prevention, Rochester, NY, United States; 3 SafeSide Australia, Brisbane, QLD, Australia; 4 Center for the Study and Prevention of Suicide, Department of Psychiatry, University of Rochester Medical Center, Rochester, NY, United States; 5 Department of Pediatrics, University of Rochester Medical Center, Rochester, NY, United States

**Keywords:** implementation science, military suicide prevention, risk formulation, safety planning, systems approach, workforce education

## Abstract

Military suicide remains an urgent global concern, with Service members facing unique stressors including frequent relocations, postings to remote and regional locations where there is a lack of external facilities, reduced social connections, extended family separations, and operational demands that heighten psychological strain. In 2021, the Australian Department of Defence (Defence) partnered with SafeSide Prevention to enhance its Suicide Prevention Program through implementation of evidence-based best practices in suicide prevention. This Community Case Study describes the rationale, implementation, and early outcomes of the Defence SafeSide Project, which aimed to embed a contemporary clinical framework and develop a system-wide approach to modernising policy, practice, and workforce education across the organisation. The partnership implemented the SafeSide Framework for Suicide Prevention, adapting it as the CARE Model (Connect, Assess, Respond, Extend) for Defence contexts. This prevention-oriented approach tailors support to each member’s circumstances through collaborative risk formulation rather than stratified risk categorisation. Major achievements included revising health and military personnel policies to remove “low”, “medium”, and “high” risk classifications, developing role-specific customised trainings, and integrating lived experience perspectives throughout implementation. Between January 2024, when the training rollout commenced, and August 2025, the national Defence workforce completed the following courses: Defence Suicide Awareness annual training (approximately 72,050 completions), CARE-Leaders and Managers (approximately 19,250 completions), CARE-Risk Formulation (approximately 1,010 completions), and CARE-Support (approximately 1600 completions). The project demonstrates how synchronised changes in policy, systems, and training can propel cultural transformation in military suicide prevention. Key lessons learned include the importance of collaborative governance structures, integration of lived experience voices, and addressing challenges in shifting from risk stratification to member-centred safety planning. While implementation is ongoing, preliminary outcomes suggest successful adoption of a prevention-oriented approach that extends suicide prevention beyond mental health settings to become a whole-of-Defence responsibility.

## 1 Introduction

Military suicide is a pressing global public health challenge, with rates among serving and ex-serving personnel rising across multiple nations (1–3). The constellation of stressors faced by military personnel – including frequent postings and deployments, separation from family and other supports, intense operational demands, a cultural emphasis on self-reliance, and complex transitions between military and civilian life – creates distinct suicide prevention challenges requiring specialised approaches (4–6).

Traditional suicide prevention approaches are typically reactive, focusing on the categorisation of risk levels of individuals with identified suicide risk. However, prevention research has increasingly challenged these approaches. Meta-analyses and systematic reviews show that stratified assessments categorising individuals as “low”, “medium”, or “high” risk are poor predictors of suicide outcomes (7–11). Furthermore, no clinical suicide risk assessment tool has sufficient predictive validity to support its exclusive use in healthcare settings. These findings have prompted a paradigm shift toward clinical judgment frameworks and prevention-oriented risk formulation (12, 13).

Rather than attempting to predict and categorise future behaviour, prevention-oriented risk formulation focuses on understanding each person’s unique context, including risk and protective factors (14–16). Clinicians gather information on foreseeable changes that are likely to affect risk and develop contingency plans for these events during safety planning. Risk is conceptualised relative to the individual’s baseline and current setting rather than population norms, enabling more personalised interventions that address specific drivers of suicidal distress while building on existing strengths and resources. This approach recognises that individuals traditionally stratified as “low risk” may still need substantial support and intervention, since a significant proportion of suicide deaths occur among this group (17).

Implementing evidence-based suicide prevention in military contexts requires attention to systems-level factors. Military organisations have unique hierarchical structures, command responsibilities, duty-of-care obligations, and operational requirements that must be considered when adapting civilian suicide prevention models. Effective military suicide prevention must balance individual autonomy with organisational responsibilities, integrate clinical and non-clinical supports, and align with military culture and values while promoting necessary shifts (18).

In 2021, the Australian Department of Defence (Defence) partnered with SafeSide Prevention to modernise its approach to suicide prevention. This collaboration aimed to update the approach to prevention from one centred on a mental health response to suicide risk to a whole-of-Defence approach – involving leadership and clinical and non-clinical personnel – grounded in contemporary evidence-based practices. This required updating clinical practices and mobilising the entire organisation to identify, respond to, and extend care for suicide risk outside health settings. The *Defence SafeSide Project* applies implementation science principles to military suicide prevention, incorporating the SafeSide Framework for Suicide Prevention. The SafeSide Framework is a map of evidence-based and best practices in suicide prevention for person-centred care (15, 19), providing a common language and standard set of skills for prevention-oriented care in service of supporting suicide prevention in systems (for a discussion of suicide prevention within systems, see Pisani & Boudreau (18)). The framework is versatile and strategic, having potential for impact at many levels of an organization. For example, it supports individual staff in case conceptualisation and best-practice care, gives supervisors and teams a shared structure for efficient review and problem-solving, helps system and quality improvement leaders evaluate and improve policies, services and pathways, and enables learning and staff development leaders to identify and address priority areas for suicide prevention education.

The SafeSide Framework was integrated into clinical practice within the Defence health system and supported with contextualised training for medical officers, psychologists, mental-health-credentialed social workers, and nurse practitioners. To enable implementation of a whole-of-organisation approach that empowered command teams and non-clinical staff to deliver member-centred responses, the framework was operationalised as the CARE Model, a unifying structure for suicide prevention training and guidelines. The CARE Model was organised around four core tasks:

- *Connect* establishes the foundation through direct inquiry about suicide, understanding the individual’s context, collaboration with support networks, and sustained commitment to safety and wellbeing (20–24).
- *Assess* structures information about stable and dynamic risk and protective factors into a prevention-oriented risk formulation (14, 15) that moves toward person-specific plans for suicide prevention (11, 16). Clinicians also identify and plan for any potential foreseeable changes that would impact a person’s safety or efficacy of the current safety plan (14, 25). For the non-clinical workforce, training focused on how they could contribute to the clinical process by sharing observations and information from their role-specific domains.
- *Respond* encompasses the range of strategies and responses that target suicidal drivers, including evidence-based treatments, mini-interventions, safety planning, means safety, observation protocols, and referrals tailored to individual needs (26–29). Staff are encouraged to tailor their selection of strategies to the person’s circumstances. Additionally, this task acknowledges that not all staff who support someone at risk are clinical staff, and yet there are still strategies that they can employ to contribute to suicide prevention efforts. Instead, a system-wide approach can offer a suite of responses to target suicidal drivers, with staff in different roles responsible for carrying out different responses.
- *Extend* ensures continuity beyond individual care episodes through caring contacts, collaborative planning, structured follow-up, access to crisis services, and warm handovers (18, 30, 31). Training emphasised practical actions to extend care beyond traditional health-care settings, recognising that meaningful support often occurs outside specialised mental health settings.

This Community Case Study describes the preparation, implementation, and early outcomes of the Defence SafeSide Project within the Exploration, Preparation, Implementation, and Sustainment (EPIS) framework (32). We detail how policy changes and customised workforce education supported the goal of strengthening evidence-based suicide prevention practices across the whole of Defence, highlighting lessons for other military and civilian contexts.

## 2 Context: the Australian Defence setting

Understanding organisational context is essential for successfully implementing systems change initiatives. The project emerged against a background of outer (e.g., sociopolitical environment, funding, interorganisational networks) and inner (e.g., organisational characteristics, leadership, protocols, workforce characteristics) contextual factors that shaped the impetus for change and the implementation approach (32).

### 2.1 Inner context

Defence encompasses full-time and reserve Australian Defence Force (Navy, Army, Air Force) members and the Department of Defence, which has a significant number of Australian Public Service (APS) personnel. Together, the workforce is approximately 100,000 personnel. Defence established its Suicide Prevention Program in the early 2000s. Until recently, clinical practice and training relied on risk stratification models that categorised members as “no”, “low”, “medium”, or “high” suicide risk, reflecting standard practice at the time. Recognising that most members do not initially present to health settings, workforce training focused on annual awareness education for all personnel.

In 2021, Defence leadership initiated the modernisation of its clinical approach to align with contemporary evidence and best practices. This included taking a more deliberate and nuanced approach to skilling the workforce to broaden responses to suicidal distress beyond traditional health settings. Key organisational strengths supporting change included governance structures, existing suicide prevention infrastructure, committed senior leadership, and a workforce accustomed to structured training and policy implementation. Challenges included deeply embedded risk stratification practices, varied understanding of suicide prevention responsibilities across roles, a workforce that prefers self-management of mental health challenges and suicidal distress, stigma associated with help-seeking, and interplay between health services and operational requirements.

### 2.2 Outer context

Several sociopolitical events catalysed the Defence SafeSide Project. In November 2020, the Interim National Commissioner for Defence and Veteran Suicide Prevention commenced an independent inquiry examining suicide deaths among serving and ex-serving Defence members. An interim report presented in Parliament in September 2021 highlighted the urgent need for systematic reform in military suicide prevention (33). The Royal Commission into Defence and Veteran Suicide was established in 2021 as an independent public inquiry to examine systemic and individual risk factors. SafeSide founder and Chief Scientific Advisor Professor Anthony Pisani provided testimony to the Commission based on his suicide prevention research and experience. The Commission released an interim report in 2022 (33) and final recommendations in 2024 (5), many of which aligned with practices Defence had already begun implementing through the SafeSide initiative. While the Defence SafeSide Project predated the Commission, it became an important component of Defence’s response to the Commission’s recommendations.

During the project period, the Australian National Suicide Prevention Strategy (34) underwent extensive consultation.^1^ The Defence SafeSide Project team monitored public releases, emerging evidence, and evolving discussions to ensure alignment with national priorities. This engagement allowed Defence to anticipate and incorporate strategic directions – including whole-of-government approaches, systems-based interventions, lived experience integration, and the shift from risk prediction towards collaborative care – before publication of the National Strategy in 2025. The National Strategy in turn validated many approaches already implemented through the Defence SafeSide Project.

In 2022, SafeWork Australia – the national workplace health and safety body – established a national policy and model codes for the Commonwealth, States, and Territories. Identification, control (prevention), and mitigation of psychosocial hazards and their sequelae are central to the policy and associated codes (see Model Work Health and Safety Bill (35)). In the context of suicide prevention, new regulations oblige employers to understand psychosocial hazards that may increase suicide risk and to introduce, maintain, and review reasonable measures to eliminate or minimise these hazards.

## 3 Preparatory work and implementation approach

### 3.1 Collaboration structure and governance

Successful implementation of system-wide change requires robust governance and clear collaboration mechanisms. The Defence SafeSide Project established a multi-tiered structure to integrate diverse perspectives throughout design and implementation. The Project Leadership Team, comprising senior representatives from Defence and SafeSide Prevention, provided overall governance, implementation oversight, and day-to-day management.

Working groups focused on policy revisions, training creation and customisation, program evaluation, implementation, and communications. Subject matter experts in each group reported to the Project Leadership Team at key milestones. The Policy Working Group reviewed policies and clinical documentation processes, developed recommendations to align with best practices, and guided revisions through to approval. The Learning Customisation Working Group oversaw adaptation of SafeSide materials for Defence contexts and the creation of additional courses for health staff, chaplaincy, wellbeing officers, leaders, and managers. The Evaluation Working Group designed data collection protocols and outcome measures. The Communications and Implementation Working Groups developed change management, stakeholder engagement, and training rollout plans.

An Advisory Group provided strategic guidance to ensure the contextual appropriateness of all outputs. This group included representatives with lived experience of suicide, command personnel across ranks and Services, and representatives from the occupational roles that would complete the training. The group’s feedback on proposed materials and implementation challenges was invaluable in navigating the balance between maintaining evidence-based practices and adapting to a complex organisation that needed to ensure operational requirements continued to be met.

The shift from risk stratification to prevention-oriented risk formulation required the development of change management strategies that supported rapid adaptation. The change management process was essential to ensure stakeholders clearly understood the changes so that anyone supporting a member could remain focused on the core task of providing member-centred support. Messaging was workshopped with the Advisory Group before rollout. Key messages emphasised that the new approach enhanced rather than replaced commitments to member safety, that all personnel retained important roles in suicide prevention regardless of clinical training, and that member-centred planning increased rather than decreased support options. Communication strategies utilised multiple channels, including command briefings, professional development sessions, email updates, intranet pages, and integration into training platforms.

### 3.2 Policy and practice transformation

#### 3.2.1 Policy review process

Comprehensive policy review and revision was a cornerstone of implementation. This review began with stakeholder consultation, including Advisory Group meetings that incorporated lived experience perspectives, collaboration with SafeSide Prevention policy experts, and in-person discussions with health personnel, military subject-matter experts, and leaders across Defence. The Policy Working Group conducted detailed analysis of 27 Defence policies and forms related to suicide prevention. An international literature review examined best practices in military and civilian suicide prevention, identifying evidence-based approaches applicable to Defence.

#### 3.2.2 Policy review outcomes and recommendations

The review identified opportunities for policy and practice enhancement. Existing policies relied on risk stratification, with specific actions tied to each risk level. Communication between health services and command was primarily triggered by assigning members to specific risk categories rather than by a tailored response to individual needs. Documentation systems reinforced categorical thinking through forms requiring designation of risk levels. Limited guidance existed for non-clinical personnel supporting at-risk members. Approaches to collaborative safety planning rarely included foreseeable changes.

The Policy Recommendation Report identified revisions that advanced best practices. These shifts were operationalised around the four CARE Model tasks. To incorporate Connect, recommendations emphasised engaging supports early and empowering personnel beyond clinicians to ask about suicide. For Assess, adopting prevention-oriented risk formulation was recommended, with documentation of individual contexts, trajectories, and foreseeable changes rather than risk categories. For Respond, guidance expanded beyond clinical interventions to include roles for command, peers, and support services in implementing member-specific strategies to support safety and recovery. Extend included recommendations for routine incorporation of non-demand caring contacts (30, 36) and clear roles for support persons in plans at members’ discretion.

#### 3.2.3 Policy updates

Changes to policy achieved three objectives across health and military personnel manuals. First, mental health professionals, medical officers, and nurse practitioners were now expected to describe risk using a prevention-oriented approach rather than stratified categories. Second, communication between health services personnel and leaders now focuses on member needs, available resources, foreseeable changes – many inclusive of operational demands – and identified supports documented in collaborative Member Safety Plans. Third, eHealth documentation for mental health risk assessments and member safety plans was updated to support practice change (see **Box 1** for examples).

Box 1 **Policy changes – before and after.**

Example 1: Risk assessment documentation.

Before: Following assessment, clinicians must assign a suicide risk level of NO FORESEEABLE, LOW, MEDIUM, or HIGH based on the presence of risk factors. Documentation must clearly state the assigned risk category.

After: Clinicians engage in collaborative risk formulation with the member, considering their unique circumstances, protective factors, and stressors. Documentation describes the formulation process and the individualised safety plan developed together with the member.

Example 2: Response protocols.

Before: HIGH risk members require hospitalisation or constant observation. MEDIUM risk members require daily monitoring and scheduled follow-up. LOW risk members may return to normal duties with routine follow-up.

After: Response is tailored to each member’s formulation and safety plan. Clinicians work collaboratively with the member to determine appropriate level of support, monitoring frequency, and care coordination based on the member’s specific needs and circumstances rather than risk category.

Example 3: Commander notification.

Before: If a member is deemed to be high risk, then a Commander must be notified to ensure appropriate workplace monitoring and duty modifications.

After: With the member’s involvement, relevant information is shared with commanders and other supports to support implementation of the safety plan. Communication focuses on specific actions and support needed rather than risk categorisation, respecting member privacy while enabling coordinated care.

These policy changes required extensive consultation to address concerns and practical challenges. A key issue involved determining what would trigger health services disclosing information to command, as high risk ratings had previously activated specific communication protocols. The solution involved developing clear guidance about circumstances warranting disclosure based on safety concerns, duty-of-care obligations, and collaborative planning with members rather than categorical risk levels. This shift required significant education efforts to ensure health personnel and command understood their roles and responsibilities under the new framework.

### 3.3 Development of role-specific customised education

The SafeSide Framework for Suicide Prevention utilises the InPlace^®^ Learning model, an educational approach that incorporates clinical expert and lived experience trainers, skills demonstrations, video-guided group learning, and interactive practice opportunities (19, 37). Previous implementations have demonstrated strong educational efficacy and perceived relevance among mental health professionals, youth services workers, and primary care trainees (19, 38). The Defence SafeSide Project required extensive customisation or creation of these materials to ensure contextual relevance and role-specific applicability across Defence.

Customisation followed a three-stage process. In Stage 1, focus groups with diverse ranks and roles identified aspects of SafeSide programs requiring adaptation, providing insights into Defence-specific language, command structures, realistic scenarios, and operational constraints. These sessions generated qualitative data about current practices, perceived barriers, and opportunities for improvement.

In Stage 2, scenario development involved gathering realistic deidentified situations from across Defence. Clinicians practicing in Defence Health Centres contributed deidentified clinical summaries. These were consolidated and presented to the Advisory Group, which voted on the most realistic and educationally valuable scenarios. The highest-scoring scenarios from each Service (Navy, Army, Air Force) were developed into demonstration scripts aligned with learning objectives. Scripts underwent review cycles with subject matter experts and lived experience representatives to ensure accuracy, sensitivity, and educational impact.

The co-designed educational content was produced in Stage 3. This included teaching with Defence instructors; filming the scenarios from Stage 2, which featured Defence personnel and professional actors; and nineteen on-camera interviews conducted with Defence members who shared professional and lived experience perspectives on suicide prevention. An extensive informed consent process, reviewed by Roses in the Ocean (Australia’s leading lived experience organisation), ensured appropriate support for participants who shared sensitive experiences. The filmed scenarios and interviews were integrated across training courses, allowing Defence voices to contribute directly to prevention education. This represented the first time many Defence members had seen colleagues openly discussing suicide prevention on screen, contributing to reduced stigma and more compassionate responses.

The customisation and creation process resulted in five trainings tailored to role-specific responsibilities: two video-guided in-person workshops and three eCourses. An enhanced Defence Suicide Awareness (DSA) annual eCourse replaced the previous Mandatory Awareness Training for all personnel. The customised DSA content introduced the CARE Model and illustrated how personnel in mental health and support roles would use it, while training strengthened the focus on prevention by exploring four key protective areas – connection, purpose, guidance, balance – that promote wellbeing and connection. The CARE Model-Leaders and Managers (CARE-LM) eCourse built on DSA to a) help command personnel and managers understand policy changes and their roles in supporting members and b) expand the four protective areas from an individual to a workplace focus. The CARE Model-Risk Formulation (CARE-RF) InPlace^®^ workshop provided intensive training for personnel with risk assessment responsibilities. The CARE Model-Documenting Risk Formulation (CARE-DRF) eCourse offered training to the same group on documentation requirements within updated eHealth systems. The CARE Model-Support (CARE-S) InPlace^®^ workshop trained health practitioners (excluding CARE-RF audiences) and non-clinical support personnel, including Chaplains and wellbeing officers, in contributing to assessment processes and providing ongoing support. The training was open to APS personnel and participation was strongly encouraged for APS staff working in Services or where it was otherwise appropriate to their responsibilities.

To support scaling of learning, each course incorporated Defence-specific content while maintaining fidelity to evidence-based practices. In the workshops, custom discussion prompts and practice exercises supported skill transfer to daily work. An additional advantage of the InPlace Learning design was that video-based modules were self-contained, reducing reliance on subject matter experts for facilitation and enhancing consistency and fidelity across the organisation. Leveraging eCourses for DSA and CARE-LM enabled rapid expansion of reach to all personnel, ensuring access to training for deployed personnel, new recruits, and geographically dispersed members. All materials met Web Content Accessibility Guidelines 2.2AA standards (39), ensuring universal access across Defence.

### 3.4 Implementation timeline and rollout strategy

Implementation followed a carefully orchestrated timeline coordinated by the Project Leadership Team and Implementation Working Group. Rollout began in January 2024 with the release of the DSA annual training, which served as both an educational tool and a medium for change management by communicating about forthcoming reforms. This sequencing allowed all personnel to be exposed to the new approach before role-specific training commenced (**Table 1**).

**Table 1:** Timeline of implementation milestones.

Date	Milestone	Description
2021	Partnership formation	Australian Department of Defence engages SafeSide Prevention to enhance Suicide Prevention Program
2022	Learning customisation	Commenced process of adapting SafeSide training and framework for Defence contexts, including creation of new training products
2022	Policy revision initiated	Review and revision of health and military personnel policies
June 2023	Policy recommendations submitted	Policy recommendations report submitted
January 2024	Training rollout begins	Launch of Defence Suicide Awareness annual training program
February – August 2024	Implementation planning	Implementation Working Group and health leaders develop detailed plans for training personnel with risk formulation responsibilities
September 2024	Training rollout continues	Release of CARE-Leaders and Managers training, prioritising command personnel who would support implementation within their units
September – October 2024	Commence training for medical officers, mental health professionals	Intensive delivery of CARE-RF workshops to medical officers, mental health professionals, and nurse practitioners
October 2024	Policy implementation	New policies formally adopted, synchronised with the completion of essential clinical training
January 2024 – August 2025	Training completion phase	72,050 completions of Defence Suicide Awareness; 19,250 completions of CARE-Leaders and Managers; 1,010 completions of CARE-Risk Formulation workshops
August 2025	Milestone achievement	Over 92,000 total training completions across all programs

Throughout implementation, multiple support mechanisms reinforced learning and addressed challenges. Regular email updates kept personnel informed of progress and upcoming changes. Question-and-answer webinars provided forums for addressing concerns and clarifying procedures. Microlearnings delivered just-in-time reminders of key concepts via email. Knowledge-share sessions formed part of continuous improvement initiatives. Alongside discussion, these included concise on-demand videos providing deeper insights into mental health assessments. For example, a short clip addressed the transition from risk stratification and focused on strategies for sharing health information with command whilst maintaining respect for member confidentiality. This multi-modal approach maximised accessibility across Defence environments while maintaining implementation momentum.

## 4 Key programmatic outcomes

Key outcomes of the Defence SafeSide Project include training reach and adoption metrics, educational outcomes, objective review of practice changes, and implementation facilitators, challenges, and adaptive responses. Ongoing collection of feedback and outcome data allows regular updates to program content with the latest suicide prevention research. All data are stored securely with access restricted to the evaluation team and key stakeholders.

Aggregate training attendance is tracked to examine reach and adoption. Feedback and outcome data are collected for all CARE Model learning experiences, supporting a data-driven approach to continuous improvement. Data collection was approved by the Departments of Defence and Veterans’ Affairs Human Research Ethics Committee. Participants voluntarily complete pre- and post-training surveys measuring knowledge, self-efficacy, attitudes toward member-centred risk formulation, and perceived utility of new procedures. Data are anonymous and matched via participant-chosen identifiers to assess changes in knowledge and attitudes over time. Facilitators, challenges, and adaptive responses to challenges are included in implementation outcomes.

### 4.1 Training reach and adoption metrics

From January 2024 through August 2025, the Defence SafeSide Project achieved substantial reach across Defence. Completion rates reflect mandatory training requirements and successful engagement strategies. **Table 2** details total course completions for each program.

**Table 2:** Training program characteristics and reach.

Training Program	Target Audience	Duration	Delivery Method	Key Learning Objectives	Completions (Jan 2024 – Aug 2025)
Defence Suicide Awareness Annual Training (DSA)	All Defence personnel	1 hour	Face-to-face presentation or Online eCourse with interactive scenarios	Recognise warning signs; understand Defence-specific stressors; know how to connect members to support; promote help-seeking culture	72,050
CARE Model: Leaders and managers supporting members at risk of suicide, self-harm, and harm to others (CARE-LM)	Military leaders, supervisors, managers	1 hour	Online eCourse with interactive scenarios	Apply CARE Model in leadership contexts; conduct supportive conversations; navigate organisational resources; create psychologically safe environments	19,250
CARE Model: Risk formulation for suicide, self-harm, and harm to others (CARE-RF)	Clinical providers, mental health professionals, medical officers, and nurse practitioners	1 day	In-person, video-guided InPlace® workshop	Conduct collaborative risk formulation; develop member-centred safety plans; move beyond risk stratification; integrate prevention-oriented assessment	1,010
CARE Model: Documenting your risk formulation (CARE-DRF)	Clinical providers, mental health professionals, medical officers, and nurse practitioners	1 hour	Online eCourse with interactive scenarios	Document a risk assessment, prevention-oriented risk formulation, and safety plan using the CARE Model in the eHealth system	980
CARE Model-Supporting members at risk of suicide, self-harm, and harm to others (CARE-S)	Other health practitioners, Chaplains, and wellbeing support personnel	4 hours	In-person, video-guided InPlace® workshop	Apply CARE Model in their roles to support members; how to share insights to help inform a prevention-oriented risk assessment conducted by mental health personnel	1600

### 4.2 Educational outcomes and preliminary impacts

Evaluation data from pre-and post-training surveys showed improvements across multiple domains (**Table 3**). As anticipated with voluntary surveys, response rates declined from pre- to post-training, although sample sizes remained notable. Average overall satisfaction was 3.96 (1–5 scale) with most participants indicating they found the training professional (86.2%) and visually engaging (79.2%). Knowledge and self-efficacy increased from pre- to post-training across all role-specific trainings. Knowledge surveys (1–6 scale) included multiple choice questions such as “What is the most effective way for you to strengthen a member’s safety plan, with member consent, in collaboration with health and wellbeing support personnel,” “Which of the following is an example of a prevention-oriented description of risk,” and “When preparing to communicate with Command about a member’s safety and support needs, what should be the primary focus of the information shared?”. Self-efficacy surveys (1–5 scale) included items such as “I feel confident in my ability to link risk assessments to member-specific safety plans,” “I feel confident in determining appropriate actions and supports for members at risk,” and “I feel confident in how to apply Defence policies in a way that considers member circumstances and context.” Most members reported that they planned to integrate what they learned in their work (74-89%) and believed the CARE Model would improve support for members (76-88%).

**Table 3:** Educational outcomes.

Domain	DSA			CARE-LM			CARE-S		CARE-RF		CARE-DRF	
	Pre		Post	Pre		Post	Pre	Post	Pre	Post	Pre	Post
	N = 13,067		N = 3,628	N = 4,510		N = 2,894	N = 965	N = 615	N = 109	N = 90	N = 326	N = 349
Knowledge (M)^a^	4.80		5.70	4.41		5.26	4.47	5.61	4.49	5.29	5.33	5.67
Self-efficacy (M)^b^	4.01		4.27	3.88		4.18	3.64	4.21	3.82	4.14	3.49	4.20
Learning transfer and Use (post-training only; percent agree or strongly agree)												
I plan to integrate what I learned into my work	86.44%			73.60%			88.63%		87.66%			
The CARE Model will improve the support we provide to members	-–			76.15%			87.95%		75.55%		-–	

DSA, Defence Suicide Awareness; CARE-LM, CARE-Leaders and Managers; CARE-S, Supporting members; CARE-RF, CARE-Risk Formulation; CARE-DRF, CARE-Documenting Risk Formulation; Pre, pre-training survey; Post, post-training survey. “I plan to integrate what I learned into my work” was asked separately for CARE-RF and DRF in 2024, then once for both in 2025. All responses were combined above.

^a^ Possible range for Knowledge survey = 1-6 (total score).

^b^ Possible range for Self-efficacy survey = 1-5 (mean score).

### 4.3 Facilitators of successful implementation

Several factors facilitated implementation. Senior leadership endorsement was crucial: visible support from Defence executive levels legitimised the initiative and encouraged participation. The Chief of the Defence Force’s public statements about suicide prevention as an organisational priority created urgency and reduced potential resistance. Senior leaders provided video footage and spoke at annual project summits to maintain leadership visibility and championship. Alignment with Royal Commission recommendations, the National Suicide Prevention Strategy, and workplace health and safety legislation provided external validation for change efforts.

The collaborative governance structure enabled rapid problem-solving while maintaining stakeholder engagement. Regular Advisory Group input ensured solutions remained practically feasible within military contexts. Targeted efforts by working groups prevented diffusion of responsibility while maintaining coordination. The Project Leadership Team’s weekly meetings enabled agile responses to emerging challenges.

Drawing on lessons learned from other projects, emphasis was placed on using existing Defence systems to support implementation, thus reducing the implementation burden. Disruption to operational activities was minimised by building on established training platforms and schedules. Technical barriers were reduced by utilising familiar eHealth systems with updated forms rather than introducing new systems. Alignment with existing quality improvement initiatives positioned the project as an enhancement rather than a replacement.

This implementation approach allowed all ranks, roles, and regions in Defence to be reached rapidly while still permitting iterative refinement to content and implementation strategy. Early DSA training created awareness and buy-in before more intensive role-specific training. Feedback from initial cohorts informed adjustments to subsequent sessions. The implementation of new policies was timed to take place concurrently with the rollout of the corresponding training, ensuring that personnel were prepared for new ways of working.

### 4.4 Implementation challenges and adaptive responses

Despite careful planning, several challenges emerged that required adaptive responses. In military settings, Commanders have significant duty-of-care obligations. In relation to suicide risk, these responsibilities were previously aligned with clear action triggers based on risk stratification. As anticipated, there was some resistance to the replacement of risk stratification with a more individualised approach to risk. Feedback indicated that structural support for new approaches, including ongoing education and policy alignment, were needed to support change. The response to this challenge included extensive consultation around how the new approach provided more nuanced guidance while still maintaining safety. In particular, specific practice examples demonstrated how prevention-oriented risk formulation could identify needs that would be missed by categorical systems.

Coordination between mental health and command is a critical element in understanding and responding to risk. Previous protocols clearly specified when and what information would be shared and what actions taken based on risk categories. The shift to prevention-oriented risk formulation required new communication guidelines. The member-centred approach requires careful balance between member privacy, command’s duty-of-care responsibilities, and operational requirements. To meet this challenge, command and health services staff were walked through scenarios in live Q&A sessions and ongoing consultation. This iterative learning process resulted in the development of supplemental resources. These helped personnel develop confidence in using prevention-oriented descriptions of risk both for communication and to drive decisions while also balancing leadership duty-of-care responsibilities and maintaining clinical judgment for complex cases.

The updating of eHealth systems to support new documentation requirements was also an iterative process. Updated forms were initially too complex, creating burdens for busy clinicians. Revisions based on user feedback resulted in streamlined formats that balanced comprehensive assessment with practical feasibility.

Defence’s large, geographically dispersed workforce and its operational tempo created access challenges that were addressed through the dual delivery of DSA in both face-to-face (where practical) and eCourse options. Feedback highlighted the importance of rapid scaling to build the internal capacity necessary to support the new approach. The need to deliver rapid education to all personnel, alongside specific messaging to command, required additional educational approaches. CARE-LM was delivered as an eCourse to align with a major health policy change that required command to understand its implications. CARE-RF and CARE-S were maintained as in-person workshops to enable targeted engagement and aligned with feedback on preference for face-to-face delivery when possible.

All programs periodically experienced technical challenges associated with limited internet bandwidth availability (e.g., audio unable to load). This was addressed for workshops through the provision of an offline download option and for eCourses with a restricted file size design. These adaptations mitigated technical challenges while preserving the intended delivery method for each component.

## 5 Discussion

The Defence SafeSide Project demonstrates the feasibility and early effectiveness of implementing system-wide suicide prevention transformation within military contexts. The shift from risk stratification to prevention-oriented risk formulation represents more than a procedural change; it reflects a fundamental reconceptualisation of how military organisations understand and respond to suicide risk. By removing categorical labels, the new approach encourages a nuanced understanding of each member’s unique situation while maintaining focus on safety and support.

High training completion rates and positive educational outcomes suggest successful knowledge transfer and attitude change across the workforce. The integration of lived experience voices appears particularly impactful, challenging stigma and normalising help-seeking within Defence’s military culture. The project’s alignment with recommendations subsequently presented by the Royal Commission validates the approach taken. Many elements implemented as parts of the Defence SafeSide Project – including movement away from risk categories, emphasis on collaborative planning, and integration of lived experience perspectives – were independently identified as priorities by the Commission, confirming that the project delivered changes seen as critical for military suicide prevention reform.

### 5.1 Lessons for military and civilian contexts

Several lessons from the Defence SafeSide Project have broader applicability (**Table 4**). First, successful system transformation requires synchronised changes across multiple domains. Policy changes would have been insufficient without accompanying training, support systems, and culture change efforts (31). Similarly, training without policy backing or health system updates would not have generated institutional support for practice change. The coordinated approach to policy, education, and implementation created mutually reinforcing changes.

**Table 4:** Key lessons learned and recommendations.

Challenge/issue	What we learned	Recommendation for others
Shifting from risk stratification mindset	Decades of low-medium-high categorisation created deeply ingrained habits among clinical and leadership personnel. Change required persistent education and policy alignment.	Begin with policy changes to create structural support for new approaches. Provide extensive training and ongoing consultation during transition period. Expect resistance and plan for iterative refinement.
Engaging senior leadership	Leadership buy-in was essential for organisation-wide adoption. Leaders needed to understand both the evidence base and practical implications for their workplace.	Invest time in leadership briefings early in process. Use data and examples relevant to military contexts. Create leadership champions at multiple organisational levels.
Integrating lived experience perspectives	Lived experience advisors provided invaluable insights that shaped training content and policy language, but integration required intentional structure. In a small community, individuals who shared their lived experience were easily recognised, leading some colleagues to approach them and their family members to discuss their mental health, inadvertently causing discomfort.	Establish formal roles and offer financial compensation in cases in which the individual is not otherwise remunerated. Create safe mechanisms for input throughout project lifecycle. Ensure diverse representation of experiences. Clarify that consent only covers using their experience as a training example, not for personal congratulations or requests for support.
Maintaining momentum across large organisation	Training 92,000+ personnel while maintaining quality and consistency required significant coordination and resource allocation.	Develop educational models to build internal capacity. Use phased rollout with clear milestones. Establish feedback mechanisms to identify and address implementation challenges quickly.
Balancing standardisation with customisation	Different Defence roles required different levels of training depth and different practical applications of the CARE Model.	Create tiered training programs matched to role requirements. Allow for local adaptation within a consistent framework. Gather feedback from diverse role groups during development.
Measuring culture change	Traditional outcome metrics (suicide rates) are insufficient for evaluating prevention-oriented culture shifts, yet stakeholders need evidence of impact.	Develop process and proximal outcome measures (help-seeking rates, safety plan completion, training satisfaction, policy compliance). Track implementation fidelity. Plan for long-term outcome evaluation.

Second, governance structures that balance expertise with stakeholder engagement are essential for navigating complex organisational contexts. The multi-tiered approach enabled technical excellence while maintaining practical feasibility. Lived experience integration enriched understanding and enhanced credibility. Command involvement ensured operational compatibility. This inclusive yet structured approach could benefit other large-scale implementation efforts.

Third, customisation for context while maintaining evidence-based core principles enables successful translation across settings. The SafeSide Framework’s fundamental elements remained intact while adapting language, scenarios, and applications for military contexts. This proactive adaptation, which balances fidelity and customisation, aligns with emerging thinking in implementation science (40) that could guide other efforts to implement evidence-based practices in specialised settings.

Fourth, addressing deeply embedded practices requires patience, consultation, and iterative refinement. The entrenchment of risk stratification thinking necessitated extensive dialogue and additional resources highlighting rationales for change and practical strategies for implementation. Providing space for concerns while maintaining momentum toward evidence-based practices proved essential. Organisations undertaking similar transformations should anticipate and plan for this change process.

### 5.2 Broader implications for suicide prevention in systems

The Defence SafeSide Project contributes to the evidence base for systems approaches to suicide prevention. By extending beyond clinical services to engage the entire organisation, it demonstrates how prevention can become embedded in organisational culture rather than remaining isolated within health services. This whole-of-system approach aligns with public health models that emphasise prevention across the continuum from universal to indicated interventions.

The successful integration of clinical and non-clinical roles suggests opportunities for similar approaches in other settings. While clinical expertise remains essential for assessment and treatment, non-clinical personnel provide crucial support for safety planning, ongoing monitoring, and social connection. This distributed model of care could help address the limited clinical workforce available while also providing more comprehensive support networks.

The emphasis on member autonomy within structured military contexts offers insights for balancing individual and organisational needs in suicide prevention. Rather than viewing these as incompatible, the project demonstrates how collaborative approaches can enhance both member agency and organisational safety responsibilities. This balance may be particularly relevant for other hierarchical organisations, such as first responder services, correctional systems, and large corporations.

### 5.3 Limitations and future directions

The evaluation timeline captures only early implementation outcomes, limiting the ability to assess sustained practice change or impacts on suicide-related outcomes. Longer-term follow-up will be essential to determine whether initial positive indicators translate into reduced suicide deaths and attempts. Because pre- and post-training surveys were voluntary, samples may be biased toward members with stronger opinions, higher engagement, or a stronger prior commitment to suicide prevention. Lower post-training response rates may also bias results toward more engaged participants. Future evaluations should explore strategies for capturing perspectives from all personnel. The focus on a single organisation limits generalisability, although lessons learned will likely transfer to other military contexts and hierarchical organisations. Comparative research across different military services or international contexts could identify universal versus context-specific elements.

Future directions for the Defence SafeSide Project include several priorities. Building on initial leadership training success, Defence will expand training for Command in 2026 by delivering rank-specific trainings that reflect the distinct challenges of early career and senior leaders. Consistent with the customisation process described above, these new trainings will include Defence instructors, interviews, and scenarios reflecting the unique contexts leaders encounter at different organisational levels. This differentiated approach demonstrates Defence’s commitment to ensuring leaders are prepared for their specific suicide prevention roles.

Impact and support for members could be expanded in multiple directions. Expansion of evaluation efforts will track longer-term outcomes, including suicide deaths, attempts, and ideation rates alongside continuous quality improvement processes. Additionally, several opportunities for expansion reflect emerging priorities in suicide prevention research. For example, the focus on the four key protective areas has been well received; a future direction includes exploring ways to deepen the development of protective social networks in alignment with emerging prevention science (41). Additionally, integrating approaches with veteran support services would ensure continuity of care during military-to-civilian transitions, a time of heightened risk. Expanding the project to include peer support programs could leverage the emphasis on mutual support in military culture. Technology-enhanced delivery methods, such as asynchronous AI-supported simulations could improve access and engagement. Exploring Restorative Just Culture approaches to postvention could further transform organisational responses following suicide incidents and deaths, further embedding a contextual, member-centred approach to understanding and responding to risk.

Research priorities emerging from this work include systematic comparison of prevention-oriented risk formulation versus stratified risk assessment in military populations. Investigation of optimal training modalities and dosage for sustaining practice change also warrants attention, while further efforts to understand the factors that influence help-seeking in military cultures could inform engagement strategies. Examination of implementation strategies across different military contexts could identify key success factors. Longitudinal studies tracking members throughout military service and transition could identify critical intervention points.

## 6 Conclusion

The Defence SafeSide Project represents a significant advance in military suicide prevention, demonstrating the feasibility of system-wide transformation from risk prediction to prevention-oriented approaches. Through synchronised policy changes, customised workforce education, and collaborative implementation strategies, Defence has begun shifting organisational culture toward member-centred, collaborative suicide prevention. While challenges remain and long-term outcomes await assessment, early indicators suggest that the adoption of evidence-based practices across the whole-of-Defence has been successful.

Moving forward, sustained commitment to implementation, careful evaluation of outcomes, and continuous improvement based on emerging evidence will be essential. The Defence SafeSide Project provides a foundation for ongoing transformation, but achieving the ultimate goal of reducing military suicide will require persistent effort, continued learning, and unwavering commitment to supporting those who serve. Other military organisations and large systems confronting suicide prevention challenges may benefit from the lessons learned through this implementation, adapting approaches to their unique contexts while maintaining focus on evidence-based, member-centred care.

## Data Availability

The datasets presented in this article are not readily available due to contractual restrictions. Access to the data may be granted on a case-by-case basis at the discretion of the Department of Defence. Requests to access the datasets should be directed to Sarah Donovan, sarah.donovan@safesideprevention.com.

## Footnotes

1 Author DM was a member of the Service Systems Working Group for the Australian National Suicide Prevention Strategy.
