# Correction: Innovating military suicide prevention: learnings from the Australian Defence SafeSide Project

**DOI:** 10.3389/fpsyt.2026.1837016

**Published:** 2026-04-20

**Authors:** Kylie Druett, Sarah Donovan, Jennifer Harvey, Dan Mobbs, Annamarie Bailey, Melanie Clark, Anthony R. Pisani

**Affiliations:** 1 Mental Health and Wellbeing Branch, Australian Department of Defence, Majura, ACT, Australia; 2 SafeSide Prevention, Rochester, NY, United States; 3 SafeSide Australia, Brisbane, QLD, Australia; 4 Center for the Study and Prevention of Suicide, Department of Psychiatry, University of Rochester Medical Center, Rochester, NY, United States; 5 Department of Pediatrics, University of Rochester Medical Center, Rochester, NY, United States

**Keywords:** implementation science, military suicide prevention, risk formulation, safety planning, systems approach, workforce education

In the **Abstract**, the month of training rollout was incorrectly listed as August 2024. This has been corrected to read: “Between January 2024, when the training rollout commenced, and August 2025, the national Defence workforce completed the following courses:”

A correction has been made to **Section 3 Preparatory work and implementation approach**, *3.1 Collaboration structure and governance*, Paragraph 3. A word was left out in error. The sentence previously stated: “This group included representatives with lived experience of suicide, command personnel across ranks and Services, representatives from the occupational roles that would complete the training.” This has been updated to read: “This group included representatives with lived experience of suicide, command personnel across ranks and Services, and representatives from the occupational roles that would complete the training.”

A correction has been made to **Section 4 Key programmatic outcomes**, *4.2 Educational outcomes and preliminary impacts*, Paragraph 1. A word was left out in error. The sentence previously stated: “Self-efficacy surveys (1–5 scale) included items as “I feel confident in my ability to link risk assessments to member-specific safety plans,” “I feel confident in determining appropriate actions and supports for members at risk,” and “I feel confident in how to apply Defence policies in a way that considers member circumstances and context.” This has been updated to read: “Self-efficacy surveys (1–5 scale) included items such as “I feel confident in my ability to link risk assessments to member-specific safety plans,” “I feel confident in determining appropriate actions and supports for members at risk,” and “I feel confident in how to apply Defence policies in a way that considers member circumstances and context.””

The **Data Availability statement** was erroneously given as “The datasets presented in this article are not readily available because Due to contractual restrictions, the dataset used in this study is not publicly available. Access to the data may be granted on a case-by-case basis at the discretion of the Department of Defence. Requests to access the datasets should be directed to Sarah Donovan, sarah.donovan@safesideprevention.com.” The correct **Data Availability statement** is “The datasets presented in this article are not readily available due to contractual restrictions. Access to the data may be granted on a case-by-case basis at the discretion of the Department of Defence. Requests to access the datasets should be directed to Sarah Donovan, sarah.donovan@safesideprevention.com.”

The **Ethics statement** was erroneously given as “Data collection was approved by Australian Government Departments of Defence and Veterans’ Affairs Human Research Ethics Committee and conducted in accordance with the local legislation and institutional requirements. Written informed consent for participation was not required in accordance with institutional requirements.” The correct **Ethics statement** is “This study was approved by Australian Government Departments of Defence and Veterans’ Affairs Human Research Ethics Committee. The study was conducted in accordance with the local legislation and institutional requirements. Written informed consent for participation was provided by the participants.”

The funder was originally only placed in the Conflict of Interest section. The **Funding** statement should read, “The author(s) declared that financial support was received for this work and/or its publication. The Australian Defence Force compensated SafeSide Prevention for programs and services provided in this project.”

The original version of this article has been updated.

